# Telehealth-Based Psychoeducation for Caregivers: The Family Intervention in Recent-Onset Schizophrenia Treatment Study

**DOI:** 10.2196/32492

**Published:** 2022-04-15

**Authors:** Kim T Mueser, Eric D Achtyes, Jagadish Gogate, Branislav Mancevski, Edward Kim, H Lynn Starr

**Affiliations:** 1 Center for Psychiatric Rehabilitation Boston University Boston, MA United States; 2 Michigan State University College of Human Medicine East Lansing, MI United States; 3 Cherry Health Grand Rapids, MI United States; 4 Janssen Research & Development, LLC Titusville, NJ United States; 5 Janssen Scientific Affairs, LLC Titusville, NJ United States

**Keywords:** schizophrenia, family psychoeducation, caregiver burden, recent-onset schizophrenia, telehealth

## Abstract

**Background:**

Schizophrenia is a lifelong illness that requires long-term treatment and caregiving. Family psychoeducation (FP) has been shown to lessen caregiver burden, improve caregiver functioning, and improve outcomes in patients. However, the impact of FP delivered specifically to caregivers on patient outcomes has not been well explored, particularly for early schizophrenia. Furthermore, there is a lack of research examining the benefits of telehealth-based psychoeducation for caregivers on either patient or caregiver outcomes.

**Objective:**

The Family Intervention in Recent-Onset Schizophrenia Treatment (FIRST) study is a randomized controlled trial of patients with schizophrenia spectrum disorders and their caregivers, which is designed to evaluate the effect of telehealth-based, caregiver-focused, study-provided psychoeducation versus usual care (UC) on patient treatment failure (TF). The impact of study-provided psychoeducation on caregiver burden is also investigated.

**Methods:**

Eligible patients and their designated caregivers were randomly assigned to either the study-provided psychoeducation (≤16 sessions of telehealth-based psychoeducation over 6 months) or UC group, stratified by antipsychotic treatment (paliperidone palmitate or oral antipsychotic). The major TF events (ie, psychiatric hospitalization or intervention, arrest or incarceration, and suicide attempts) were assessed at 3, 6, and 12 months after baseline. A proportional means model using mean cumulative function was used to assess between-group differences in the mean cumulative number of TF events over 12 months. Caregiver burden was assessed using the Involvement Evaluation Questionnaire and 12-item Short Form Health Survey.

**Results:**

A total of 148 pairs of participants were enrolled in the study, of whom 96 (64.9%) patients and 94 (63.5%) caregivers completed the 12-month follow-up. The mean number of sessions in the study-provided psychoeducation group was 7.7 (SD 5.9). No differences were observed between the study-provided psychoeducation and UC groups in patient outcomes (rates of TF: 70% vs 67%; *P*=.90) or measures of caregiver burden (assessment of caregiver distress and physical and mental health). However, post hoc analyses revealed lower relapse rates in patients who received paliperidone palmitate than in those who received oral antipsychotics at all time points. Although the FIRST study did not meet the primary end point, several key lessons were identified to inform future caregiver-focused, telehealth-based FP interventions. Lack of study-provided psychoeducation, focus on caregiver-only intervention, difficulties with enrollment, and caregiver–treatment team coordination may have affected the outcomes of the FIRST study.

**Conclusions:**

Key insights from the FIRST study suggest the potential importance of supporting sufficient caregiver engagement; communication between clinicians, patients, and family members regarding treatment plans; and solidifying the relationship between clinicians providing psychoeducation to the caregiver and patient treatment team.

**Trial Registration:**

ClinicalTrials.gov NCT02600741; http://clinicaltrials.gov/ct2/show/NCT02600741

## Introduction

Schizophrenia is a complex, lifelong illness that typically develops in young adults [1] and requires long-term treatment and caregiving, which are frequently provided by family members [2,3]. Caregivers often find that caring for a loved one with schizophrenia is difficult and struggle with social isolation, financial burden, and physical and emotional exhaustion [4,5]. Family psychoeducation (FP), a guideline-recommended complement to pharmacological treatment for schizophrenia, has been shown to lower burden and improve functioning in caregivers and can also lead to improved patient outcomes, including lower rates of relapse and hospitalization [6-11]. However, FP is often unavailable or underused, partially because of implementation barriers such as scheduling difficulties and lack of access to care from specialists [12-15].

To address this unmet need, web-based or telehealth-based models of psychoeducation that offer private at-home sessions have been developed [16-18]. Compared with usual care (UC), web-based FP interventions involving caregiver support, patient psychoeducation, and mutual patient–caregiver support have been found to be successful in lowering stress, reducing symptoms, increasing perceived social support for patients with schizophrenia, and improving the illness knowledge of caregivers [19,20]. Family interventions during the early phase of illness have been studied; however, the efficacy of FP interventions delivered exclusively to caregivers is still being explored.

The Family Intervention in Recent-Onset Schizophrenia Treatment (FIRST) study was designed to evaluate the impact of FP given specifically to caregivers on the outcomes of patients with schizophrenia spectrum disorder under their care and family burden. In the FIRST study, FP was delivered using Healios Inc, doing business as MyHealios, a telehealth-based study-provided psychoeducation (SPPE) and skills training intervention. MyHealios was developed to incorporate common components of efficacious caregiver-oriented FP interventions during the patients’ early phase of illness; the FP program was individualized to each caregiver to include education about schizophrenia and its treatment and skills training to improve communication, problem solving, and coping [21-23]. The MyHealios live web-based sessions were clinician led, enabling caregivers to access professional services from home. This paper reports the primary findings of the FIRST study and outlines other key learnings of the study.

## Methods

### Study Design and Patients

**Figure 1:**
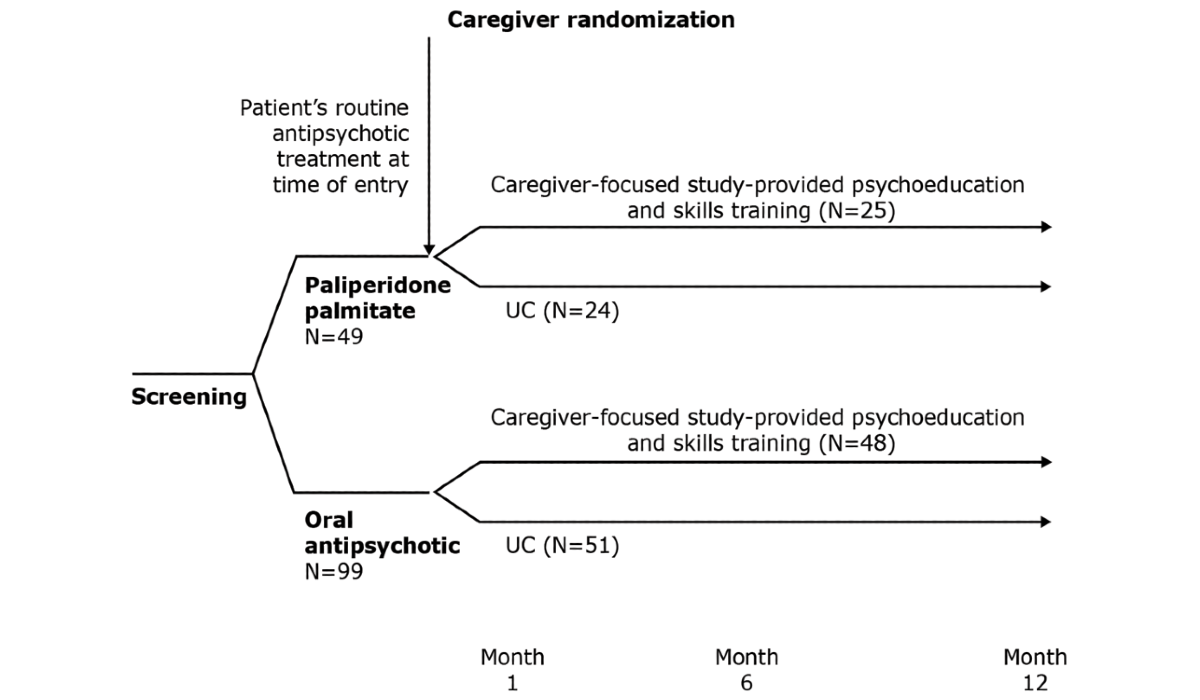
FIRST study design. In the FIRST study, caregivers randomized to the study-provided psychoeducation received up to 16 study-provided psychoeducation and skills training sessions within a 6-month period. UC consisted of caregiver support that was customarily provided by the study site (if any). FIRST: Family Intervention in Recent-Onset Schizophrenia Treatment; UC: usual care.

The FIRST study (NCT02600741) was a randomized controlled trial of patients with schizophrenia spectrum disorders and their caregivers, that was conducted to evaluate the overall effect of caregiver-focused study-provided psychoeducation and skills training compared with UC on the number of treatment failure (TF) events in patients (Multimedia Appendix 1). The study design was informed by a meta-analysis of caregiver-directed psychosocial interventions [24]. The FIRST study was initiated on July 24, 2015, and completed on July 5, 2018. The study sites were 31 community mental health centers in the United States, which provide routine clinical care to patients with schizophrenia. The study investigators received formal training through an investigator meeting and other training provided by the sponsor. Study participants were patients with diagnoses of schizophrenia, schizoaffective disorder, or schizophreniform disorder, aged 18 to 35 years, who were receiving paliperidone palmitate or oral antipsychotics, as prescribed by their clinician. Participants must have had ≥1 TF within 6 months of screening, defined as psychiatric hospitalization, intensive outpatient psychiatric treatment or partial hospitalization, psychiatric emergency department visit, crisis center visit, mobile crisis unit intervention, arrest or incarceration, or suicide attempt. Caregivers were individuals who provided the patient with assistance and care. They could be members of the immediate or extended family, friends, neighbors, or significant others. Caregivers were included if they were aged ≥18 years, had verbal interaction with the patient ≥2 times a week, had internet access, and had not received formal psychoeducation in the past 12 months. After screening, caregivers were randomly assigned in a 1:1 ratio to the study-provided psychoeducation or UC, stratified by patient antipsychotic treatment (paliperidone palmitate or oral antipsychotic; Figure 1). If the caregiver was unable or unwilling to continue participation in the study, the caregiver was not replaced; however, the patient was followed up. If a patient withdrew from the study, both the patient and caregiver were discontinued from the study.

### Ethical Considerations

The study protocol was approved by an institutional review board (ID #5146C) and conducted in accordance with the Declaration of Helsinki and was consistent with Good Clinical Practices and applicable regulatory requirements. Patients and their legally acceptable representatives provided written informed consent. Further details of the study design can be accessed on the ClinicalTrials.gov page for the FIRST study [25].

### Interventions

Caregivers randomly assigned to the study-provided psychoeducation group were invited to attend up to 16 live web-based sessions of MyHealios, a telehealth-based FP and skills training program for caregivers of patients with schizophrenia over a 6-month period. Each caregiver was assigned a trained and certified masters-level clinician who was independent of, and had no communication with, the patient’s UC team. All MyHealios clinicians received formal training and a training manual. They also underwent a certification process before conducting FP sessions with the caregivers enrolled in the study. The clinician who developed the FP curriculum and supervised the caregiver sessions was a PhD-level clinical psychologist with expertise in FP interventions for schizophrenia. Regular (eg, weekly) supervision was provided to the certified clinicians throughout the study. The adherence of clinicians to the FP and skills training program and manual was routinely evaluated using a 10-point fidelity scale based on observations of recorded caregiver sessions (including items such as agenda setting, collaboration, efficient use of time, interpersonal effectiveness, and following the structure of skills training), with fidelity ratings provided as feedback to clinicians and incorporated into supervision.

The MyHealios clinicians worked with the caregivers through live web-based sessions on a one-on-one basis throughout the program. Each session was 40 minutes in length and was conducted on the web at a time convenient for the caregiver. The web interface included live videos of both the caregiver and clinician, as well as a chat window to facilitate communication and caregiver participation in interactive activities. The number of delivered sessions and topics were determined jointly by the caregiver and clinician, with the teaching information and skills individually tailored to the caregiver. During each session, the caregiver presented problems that arose from caring for the patient and elaborated with specific examples. The clinician offered training and guidance on the appropriate methods to manage the identified problems.

Sessions were planned to occur weekly at the beginning of the program and decrease in frequency over the next 6 months as participants learned how to apply the skills in their day to day lives. A total of 3 modules were identified for initial completion by all caregivers (engagement and goal setting, communications, problem solving and goal achievement). Caregivers could then elect to complete any of the other modules in any order (coping, relapse prevention, delusions, low levels of activity, schizophrenia, anxiety, bipolar disorder, hallucinations, crisis identification and management, alcohol and drugs, depression, engaging the treatment team, and treatment adherence).

The UC group received support routinely provided by caregivers at the study sites. In both groups, patients and their caregivers were followed up for ≤12 months after the baseline assessment.

### Assessments

Assessments, including those of TF events, were evaluated at baseline and at 3, 6, and 12 months. Patient illness self-management was evaluated using the self-reported Illness Management and Recovery (IMR) scale [26]. This self-reported scale contains 15 questions, each of which is answered on a 5-point Likert scale, with higher scores indicating better recovery status. The IMR total score (range 15-75) was derived as the sum of the 15 item scores. The severity of psychotic symptoms was rated using the Clinical Global Impression of Severity (CGI-S) scale [27] by a member of the patient’s treatment team (not a family clinician) who was not masked to the treatment assignment. The CGI-S rating scale rates the severity of a participant’s psychotic condition based on a 7-point global assessment of symptom severity from 1 (normal, not ill) to 7 (most extremely ill).

Caregiver-reported assessments were conducted at the same time as patient assessments. The Involvement Evaluation Questionnaire (IEQ) [28] was used to measure caregiver distress, and the 12-item Short Form Health Survey (SF-12) [29] was used to measure overall perceived physical health (physical component score [PCS]) and mental health (mental health component score [MCS]). The IEQ is designed to measure the consequences of caregiving on family members and friends of patients with schizophrenia. All items are scored on a scale of 0 (never) to 4 (always), and the total score ranges from 0 to 108. Higher IEQ scores indicate higher levels of caregiver burden. The SF-12 is a self-administered 12-item questionnaire designed to cover 8 domains of functional health status and well-being: physical functioning, role limitations because of physical health problems, bodily pain, general health perceptions, vitality, social functioning, role limitations because of emotional problems, and mental health. These scales are scored from 0 to 100, with higher scores indicating better health. A 1-week recall period was used for PCS and MCS.

Safety was assessed based on reported adverse events (AEs) and serious AEs (SAEs). AEs and SAEs were reported for patients, and only SAEs were reported for caregivers.

Medical resource utilization, including hospitalizations, emergency department visits, and outpatient services for patients and caregivers, was recorded on a patient health resource utilization form by chart abstraction and an interview or questionnaire if data were missing.

### Statistical Analyses

The primary efficacy end point was the mean cumulative number of TF events experienced by patients over the 12-month study period. A proportional means model using the mean cumulative function was used to assess between-group differences in the mean cumulative number of TF events over 12 months. The mean cumulative function, as a function of time, was defined as the expected (mean) number of TF events in a given time interval since study day 1. The mean cumulative function for recurrent events and Kaplan–Meier (for time to the first event) analyses were performed for overall TF because of any event and for TF because of each of the events specified in the definition of TF. For secondary outcomes, changes from baseline to 3, 6, and 12 months in IEQ, IMR, SF-12, and CGI-S scores were analyzed using a mixed model repeated measures methodology with terms for study group, time, study group by time interaction, and baseline score. In addition, treatment-emergent AEs (TEAEs) were presented according to the treatment group (defined by the antipsychotic medication at baseline: paliperidone palmitate or oral antipsychotics).

The TF rate in the control group was assumed to be 0.50 based on a previous study with a similar end point [30]. The effect size in terms of a risk ratio of 0.60 was obtained from a meta-analysis of 18 randomized controlled studies examining the effect of face-to-face psychoeducation for caregivers on similar end points [24].

## Results

### Disposition

**Figure 2:**
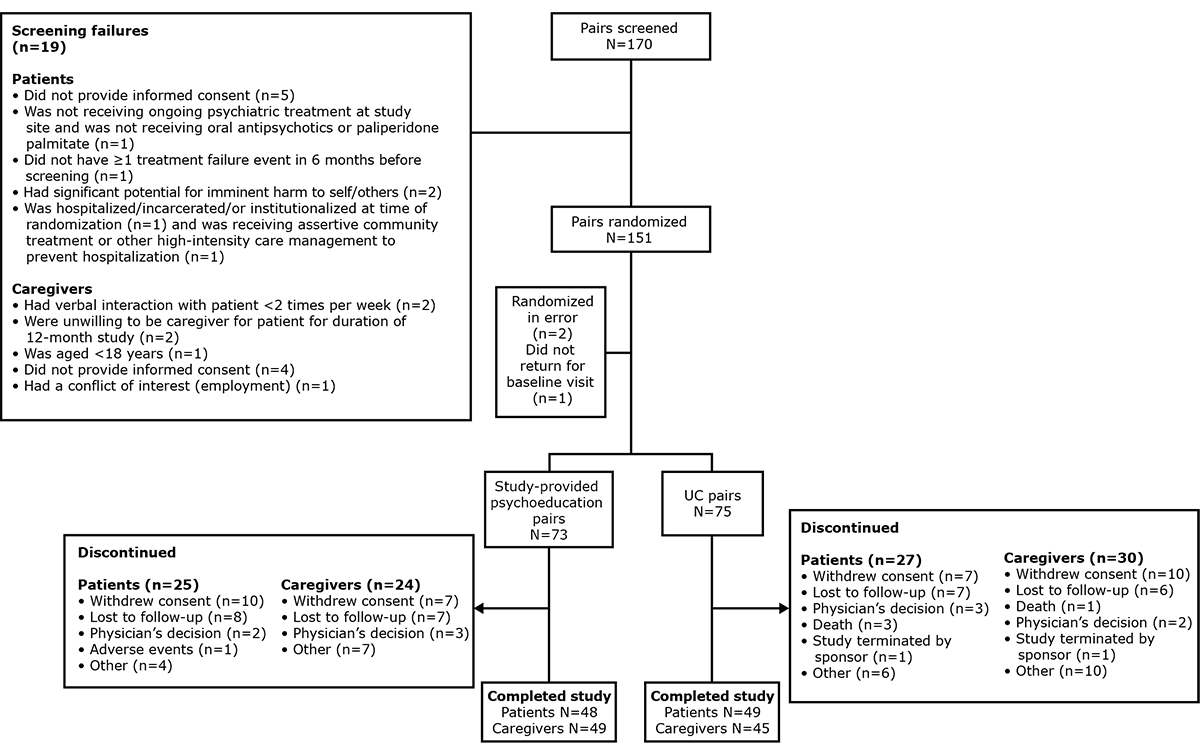
Disposition of study pairs in the FIRST study. Study patient pairs comprised individuals with schizophrenia and their designated caregivers. Patients could have ≥1 reason for screen failure. FIRST: Family Intervention in Recent-Onset Schizophrenia Treatment; UC: usual care.

Owing to difficulties in study enrollment, recruitment was discontinued before the target enrollment of 300 pairs was met, resulting in underpowered statistical analyses. A total of 170 patient–caregiver pairs were screened in the study; 19 pairs had screening failures (some with more than 1 reason for a total of 21 screening failures [Figure 2]). As a result, 151 (88.8%) were randomly assigned to study-provided psychoeducation or UC; of these 151 pairs, 148 (98%) patient–caregiver pairs were included in the all-randomized analysis set (study-provided psychoeducation, n=73, 49.3%; UC, n=75, 50.7%). Of the 148 participants, 96 (64.9%) patients and 94 (63.5%) caregivers completed 12 months of follow-up; 52 (35.1%) patients and 54 (36.5%) caregivers discontinued participation before 12 months (Figure 2).

### Demographics and Baseline Characteristics

Patients’ and caregivers’ demographics and baseline characteristics were generally balanced across the study-provided psychoeducation and UC groups (Tables 1 and 2). The median patient age was 25.0 (range 18 to 35) years, suggesting that patients were early in their disease course; most patients were male (111/148, 75%), White (84/148, 56.8%), and living with family or friends (131/148, 88.5%). Of the 148 participants, at baseline, 49 (33.1%) patients were receiving paliperidone palmitate, and 99 (66.9%) were receiving oral antipsychotics. The mean CGI-S score was 4.2, indicating moderate severity of illness (Table 1). The median caregiver age was 52.5 (range 21-76) years, with most being female (116/148, 78.4%), White (87/148, 58.8%), and a parent of the patient (112/148, 75.7%). Baseline IEQ total scores, SF-12 PCS scores, and SF-12 MCS scores were similar in the study-provided psychoeducation and UC groups (Table 1).

Caregivers who discontinued participation early tended to be nonparent relatives with lower self-reported health on the SF-12 and high burden scores on the IEQ at baseline (Table 2). The baseline demographic and clinical characteristics of the patients whose caregivers discontinued participation by month 12 were similar.

**Table 1:** Demographics and baseline characteristics of patients with schizophrenia spectrum disorders and caregivers by study group (N=148).

Parameter				Intervention^a^				
				Study-provided psychoeducation (n=73)		Usual care (n=75)		Total
**Patients**								
	Age (years), mean (SD)		25.3 (4.7)		25.1 (5.0)		25.2 (4.8)	
	**Sex, n (%)**							
		Male	54 (74)		57 (76)		111 (75)	
		Female	19 (26)		18 (24)		37 (25)	
	**Race, n (%)**							
		White	45 (61.6)		39 (52)		84 (56.8)	
		Black or African American	21 (28.8)		31 (41.3)		52 (35.1)	
		Multiple or other	7 (9.6)		4 (5.3)		11 (7.4)	
		Unknown or not reported	0 (0)		1 (1.3)		1 (0.7)	
	**Ethnicity, n (%)**							
		Hispanic or Latino	13 (17.8)		17 (22.7)		30 (20.3)	
		Not Hispanic or Latino	60 (82.2)		57 (76)		117 (79.1)	
		Unknown or not reported	0 (0)		1 (1.3)		1 (0.7)	
	**Living status, n (%)**							
		At home with family or friends	63 (86.3)		68 (90.7)		131 (88.5)	
		At home alone	5 (6.8)		5 (6.7)		10 (6.8)	
		Sheltered living	2 (2.7)		0 (0)		2 (1.4)	
		Other	3 (4.1)		1 (1.3)		4 (2.7)	
	**Diagnosis, n (%)**							
		Schizophrenia	44 (60.3)		38 (50.7)		82 (55.4)	
		Schizoaffective disorder	31 (42.5)		34 (45.3)		65 (43.9)	
		Schizophreniform disorder	0 (0)		5 (6.7)		5 (3.4)	
	**Functioning, mean (SD)**							
		IMR^b^ total score	48.3 (6.8)		49.2 (7.1)		48.8 (7.0)	
		CGI-S^c^ score	4.1 (1.1)		4.3 (1.1)		4.2 (1.1)	
**Caregivers**								
	Age (years), mean (SD)		52.1 (11.2)		49.0 (12.5)		50.5 (11.9)	
	**Sex, n (%)**							
		Male	14 (19.2)		18 (24)		32 (21.6)	
		Female	59 (80.8)		57 (76)		116 (78.4)	
	**Race, n (%)**							
		White	47 (64.4)		40 (53.3)		87 (58.8)	
		Black or African American	18 (24.7)		31 (41.3)		49 (33.1)	
		Multiple or other	7 (9.6)		2 (2.7)		9 (6.1)	
		Not reported or unknown	1 (1.4)		2 (2.7)		3 (2.0)	
	**Ethnicity, n (%)**							
		Hispanic or Latino	9 (12.3)		16 (21.3)		25 (16.9)	
		Not Hispanic or Latino	64 (87.7)		58 (77.3)		122 (82.4)	
		Not reported	0 (0)		1 (1.3)		1 (0.7)	
	**Relationship with patient, n (%)**							
		Parent^d^	56 (76.7)		56 (74.7)		112 (75.7)	
		Sibling	2 (2.7)		6 (8.0)		8 (5.4)	
		Other relative	5 (6.8)		4 (5.3)		9 (6.1)	
		Spouse or partner	6 (8.2)		5 (6.7)		11 (7.4)	
		Friend	3 (4.1)		2 (2.7)		5 (3.4)	
		Other	1 (1.4)		2 (2.7)		3 (2.0)	
	**IEQ^e^ score, mean (SD)**							
		Total	30.8 (16.4)		29.7 (17.3)		30.2 (16.8)	
		Tension	8.1 (5.4)		6.8 (6.0)		7.5 (5.7)	
		Supervision	3.1 (3.3)		3.8 (3.8)		3.5 (3.6)	
		Worrying	11.3 (6.2)		9.7 (5.8)		10.5 (6.0)	
		Urging	10.7 (5.9)		11.9 (7.1)		11.3 (6.6)	
	SF-12^f^ PCS^g^ score, mean (SD)^h^		50.3 (10.3)		51.7 (9.4)		51.0 (9.8)	
	SF-12 MCS^i^ score, mean (SD)^h^		45.3 (10.4)		48.7 (9.5)		47.0 (10.1)	

^a^All-randomized analysis set (all caregivers or patients who were randomly assigned and entered the study).

^b^IMR: Illness Management and Recovery.

^c^CGI-S: Clinical Global Impression of Severity.

^d^Includes stepparents, foster parents, and adoptive parents.

^e^IEQ: Involvement Evaluation Questionnaire.

^f^SF-12: 12-item Short Form Health Survey.

^g^PCS: physical component summary.

^h^For SF-12 (PCS and MCS), there were 43 caregivers in the discontinued early group, 102 in the completed study group, and 145 in the total group.

^i^MCS: mental component summary.

**Table 2:** Demographics and baseline characteristics of patients with schizophrenia spectrum disorders and caregivers by caregiver discontinuation status (N=148).

Parameter					Caregiver discontinuation status^a^						
					Discontinued early (n=45)			Completed study (n=103)			Total
**Patients**											
	Age (years), mean (SD)			25.2 (4.3)			25.2 (5.1)			25.2 (4.8)	
	**Sex, n (%)**										
		Male	31 (68.9)			80 (77.7)			111 (75)		
		Female	14 (31.1)			23 (22.3)			37 (25)		
	**Race, n (%)**										
		White	26 (57.8)			58 (56.3)			84 (56.8)		
		Black or African American	17 (37.8)			35 (34.0)			52 (35.1)		
		Multiple or other	1 (2.2)			10 (9.7)			11 (7.4)		
		Unknown or not reported	1 (2.2)			0 (0)			1 (0.7)		
	**Ethnicity, n (%)**										
		Hispanic or Latino	8 (17.8)			22 (21.4)			30 (20.3)		
		Not Hispanic or Latino	37 (82.2)			80 (77.7)			117 (79.1)		
		Unknown or not reported	0 (0)			1 (1.0)			1 (0.7)		
	**Living status, n (%)**										
		At home with family or friends	41 (91.1)			90 (87.4)			131 (88.5)		
		At home alone	3 (6.7)			7 (6.8)			10 (6.8)		
		Sheltered living	0 (0)			2 (1.9)			2 (1.4)		
		Other	1 (2.2)			3 (2.9)			4 (2.7)		
	**Diagnosis, n (%)**										
		Schizophrenia	29 (64.4)			53 (51.5)			82 (55.4)		
		Schizoaffective disorder	19 (42.2)			46 (44.7)			65 (43.9)		
		Schizophreniform disorder	0 (0)			5 (4.9)			5 (3.4)		
	**Functioning, mean (SD)**										
		IMR^b^ total score, mean (SD)	46.0 (6.2)			50.0 (7.0)			48.8 (7.0)		
		CGI-S^c^ score, mean (SD)	4.2 (1.0)			4.2 (1.2)			4.2 (1.1)		
**Caregivers**											
	Age (years), mean (SD)			47.8 (13.3)			51.7 (11.2)			50.5 (11.9)	
	**Sex, n (%)**										
		Male	9 (20)			23 (22.3)			32 (21.6)		
		Female	36 (80)			80 (77.7)			116 (78.4)		
	**Race, n (%)**										
		White	28 (62.2)			59 (57.3)			87 (58.8)		
		Black or African American	16 (35.6)			33 (32)			49 (33.1)		
		Multiple or other	0 (0)			9 (8.7)			9 (6.1)		
		Not reported or unknown	1 (2.2)			2 (1.9)			3 (2)		
	**Ethnicity, n (%)**										
		Hispanic or Latino	7 (15.6)			18 (17.5)			25 (16.9)		
		Not Hispanic or Latino	38 (84.4)			84 (81.6)			122 (82.4)		
		Not reported	0 (0)			1 (1)			1 (0.7)		
	**Relationship with patient, n (%)**										
		Parent^d^	29 (64.4)			83 (80.6)			112 (75.7)		
		Sibling	4 (8.9)			4 (3.9)			8 (5.4)		
		Other relative	5 (11.1)			4 (3.9)			9 (6.1)		
		Spouse or partner	4 (8.9)			7 (6.8)			11 (7.4)		
		Friend	2 (4.4)			3 (2.9)			5 (3.4)		
		Other	1 (2.2)			2 (1.9)			3 (2)		
	**IEQ^e^ score, mean (SD)**										
		Total	33.2 (18.2)			29.0 (16.1)			30.2 (16.8)		
		Tension	8.6 (6.1)			7.0 (5.6)			7.5 (5.7)		
		Supervision	4.2 (4.6)			3.1 (3.0)			3.5 (3.6)		
		Worrying	10.7 (6.2)			10.5 (6.0)			10.5 (6.0)		
		Urging	12.1 (7.2)			10.9 (6.2)			11.3 (6.6)		
	SF-12^f^ PCS^g^ score, mean (SD)^h^			48.5 (11.4)			52.0 (8.9)			51.0 (9.8)	
	SF-12 MCS^i^ score, mean (SD)^h^			46.4 (10.2)			47.2 (10.0)			47.0 (10.1)	

^a^Safety analysis set (all caregivers or patients who entered the study).

^b^IMR: Illness Management and Recovery.

^c^CGI-S: Clinical Global Impression of Severity.

^d^Includes stepparents, foster parents, and adoptive parents.

^e^IEQ: Involvement Evaluation Questionnaire.

^f^SF-12: 12-item Short Form Health Survey.

^g^PCS: physical component summary.

^h^For SF-12 (PCS and MCS), there were 43 caregivers in the discontinued early group, 102 in the completed study group, and 145 in the total group.

^i^MCS: mental component summary.

### Extent of Exposure to Caregiver Support and Education Program

In the study-provided psychoeducation group, the mean number of caregiver sessions received was 7.7 (SD 5.88), and the median was 8 (range 0-16). Of the 73 participants, 40 (55%) caregivers who were randomly assigned to the study-provided psychoeducation intervention group received at least half of the modules (ie, ≥8 sessions); 12 (16%) caregivers did not receive any sessions, and 7 (10%) caregivers received only 1 session; 9 (12%) caregivers received 15 training sessions, and 3 (4%) received a maximum of 16 sessions (Multimedia Appendix 2). Of the 73 participants, 61 (84%) caregivers received at least one session, of whom all (n=73, 100%) received the *engagement and goal setting* module, 52 (85%) received the *communications* module, 40 (66%) received the *problem solving and goal achievement* module, and 35 (57%) received the *coping* module. The other modules were assigned to <50% of the caregivers (Tables 3 and 4). Caregivers who received fewer sessions were younger and more likely to be spouses or partners than those who received more sessions (Multimedia Appendix 3). Of the 75 caregivers in the UC group, 59 (79%) received no support services, and 7 (9%) were provided with case management or individual counseling or therapy.

**Table 3:** Summary of different modules administered to caregivers during study-provided psychoeducation (N=73)^a^.

Caregiver-focused study-provided psychoeducation module description	Paliperidone palmitate (n=25^b^), n (%)	Oral antipsychotics (n=48^b^), n (%)	Total, n (%)
Engagement and goal setting	22 (100)	39 (100)	61 (100)
Communications	18 (82)	34 (87)	52 (85)
Problem solving and goal achievement	15 (68)	25 (64)	40 (66)
Coping	13 (59)	22 (56)	35 (57)
Release prevention	8 (36)	10 (26)	18 (30)
Delusions	7 (32)	10 (26)	17 (28)
Low levels of activity	3 (14)	9 (23)	12 (20)
Schizophrenia	3 (14)	7 (18)	10 (16)
Anxiety	4 (18)	5 (13)	9 (15)
Bipolar	3 (14)	2 (5)	5 (8)
Hallucinations	3 (14)	2 (5)	5 (8)
Crisis identification and management	2 (9)	2 (5)	4 (7)
Alcohol and drugs	0 (0)	3 (8)	3 (5)
Depression	0 (0)	2 (5)	2 (3)
Engaging the treatment team	1 (5)	0 (0)	1 (2)
Treatment adherence	0 (0)	1 (3)	1 (2)

^a^All-randomized analysis set (all caregivers who were randomly assigned and entered the study).

^b^A total of 12 caregivers (paliperidone palmitate, n=3; oral antipsychotics, n=9) did not receive any modules; percentages are given as a proportion of the caregivers receiving modules.

**Table 4:** Summary of different modules administered to caregivers during caregiver support in usual care (N=75)^a^.

Usual care provided	Paliperidone palmitate (n=24), n (%)	Oral antipsychotics (n=51), n (%)	Total, n (%)
None	21 (88)	38 (75)	59 (79)
Case management	1 (4)	6 (12)	7 (9)
Individual counseling or therapy	1 (4)	6 (12)	7 (9)
NAMI^b^	2 (8)	3(6)	5 (7)
Group counseling or therapy	0 (0)	2 (4)	2 (3)
Option to join NAMI family-to-family education program	0 (0)	1 (2)	1 (1)
Live interaction	0 (0)	1 (2)	1 (1)
Supportive therapy	0 (0)	1 (2)	1 (1)
Website link	0 (0)	1 (2)	1 (1)

^a^All-randomized analysis set (all caregivers who were randomly assigned and entered the study).

^b^NAMI: National Alliance on Mental Illness.

### Efficacy

#### TF Events

**Figure 3:**
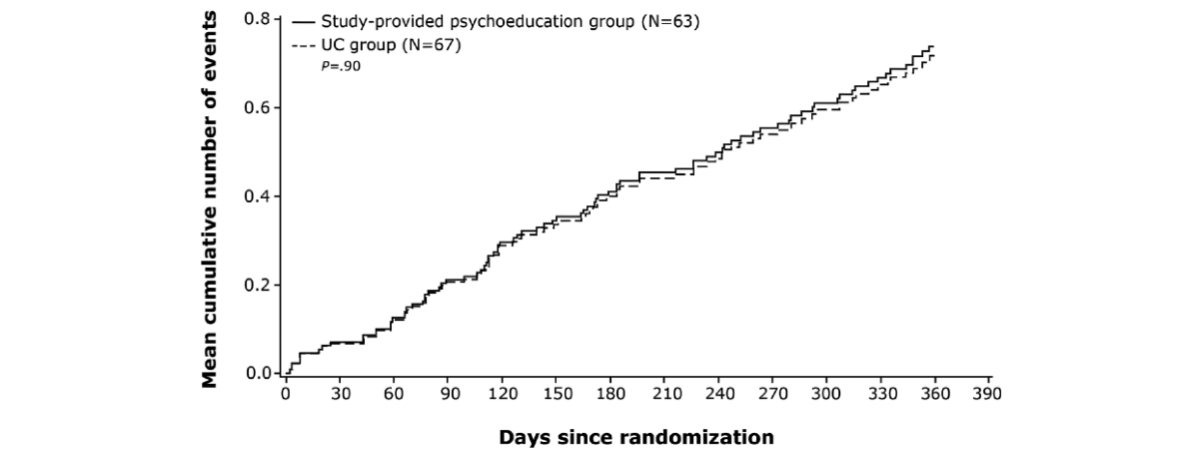
Cumulative mean functions of treatment failure because of any event in the study-provided psychoeducation and UC groups UC: usual care.

**Figure 4:**
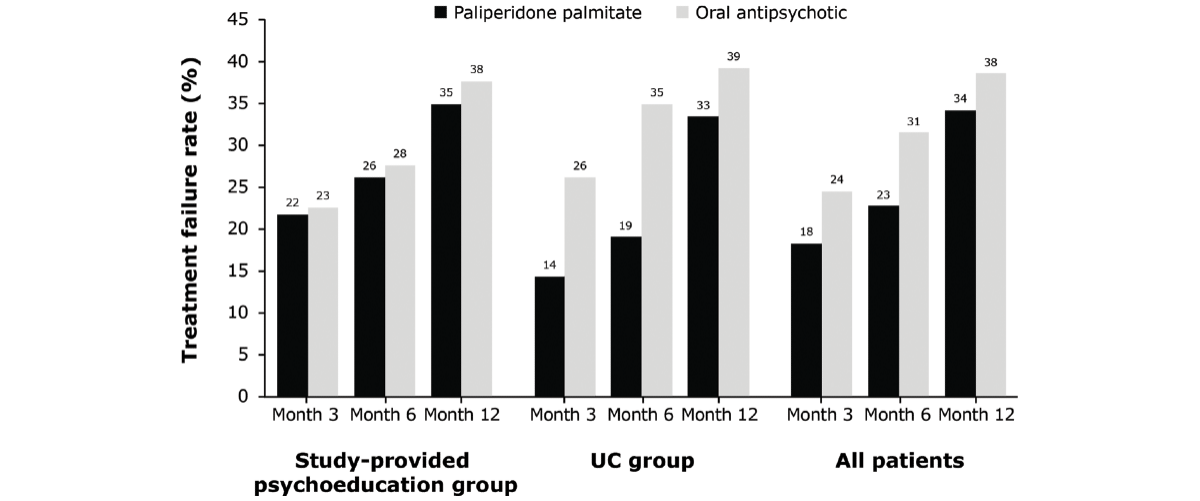
Treatment failure rates by antipsychotic treatment strata (post hoc analysis). Efficacy analysis set (n=130, all patients who entered the study and had at least one postbaseline efficacy assessment). UC: usual care.

A total of 89 TF events occurred during the study. Approximately 37% (23/63) of participants in the study-provided psychoeducation group and 37% (25/67) of participants in the UC group had at least 1 TF due to any event. TF rates were not associated with baseline CGI-S scores and did not differ between the study-provided psychoeducation and UC groups (*P*=.90; Figure 3). Most TF events were because of psychiatric hospitalization (61/89, 69%) or psychiatric emergency department visits (13/89, 15%). Post hoc analyses also showed lower relapse rates in patients who received paliperidone palmitate than in those who received oral antipsychotics at all time points (Figure 4).

Exploratory post hoc analyses were performed to investigate whether higher levels of caregiver participation in the study-provided psychoeducation intervention were associated with improved patient TF outcomes. There was no significant difference in the mean number of TFs because of any event between caregivers who received >8 sessions versus the overall UC group (36% vs 37%; *P*=.76). In the study-provided psychoeducation group, TF rates were notably higher in patients whose caregivers received at least one session than in patients of caregivers who received 15 to 16 sessions (10/13, 77% vs 4/12, 33%; Table 5).

**Table 5:** Summary of secondary end points: caregiver and patient secondary outcomes.

Training sessions	Number of participants, N	Patients with ≥1 treatment failure, n (%)	Total treatment failures, n	Treatment failure rate
Total	63	23 (36.5)	44	0.70
0-1	13	5 (38.5)	10	0.77
2-14	38	15 (39.5)	30	0.79
15-16	12	3 (25)	4	0.33

#### Secondary Outcomes

Caregiver IEQ total scores, SF-12 PCS and MCS scores, patient IMR total scores, and CGI-S scores improved from baseline to the follow-up assessments for both the study-provided psychoeducation and UC groups (Tables 6-8). However, there were no statistically significant differences in change from baseline between groups at any time point (*P*>.05 for all comparisons). Similar decreases from baseline in health resource use at months 6 and 12 were observed in the study-provided psychoeducation and UC groups.

**Table 6:** Summary of secondary end points: health resource utilization outcomes.

Outcomes					3 months					6 months					12 months		
					Study-provided psychoeducation		UC^a^		Study-provided psychoeducation			UC		Study-provided psychoeducation			UC
**Caregiver outcomes**																	
	**IEQ^b,c^ change from baseline**																
		LS^d^ mean (SE)		−2.95 (1.59)		−5.08 (1.54)		−6.96 (1.59)			−7.62 (1.58)		−4.15 (1.96)			−5.61 (2.08)	
		**Difference (study-provided psychoeducation vs UC)**															
			LS mean (95% CI)	2.13 (−2.25 to 6.52)		2.13 (−2.25 to 6.52)		0.66 (−3.79 to 5.10)			0.66 (−3.79 to 5.10)		1.46 (−4.21 to 7.13)			1.46 (−4.21 to 7.13)	
			*P* value	.34		.34		.77			.77		.61			.61	
	**SF-12^e^ MCS^f,g^ change from baseline**																
		LS mean (SE)		0.63 (1.16)		1.27 (1.12)		2.86 (1.07)			2.92 (1.07)		1.94 (1.34)			3.01 (1.42)	
		**Difference (study-provided psychoeducation vs UC)**															
			LS mean (95% CI)	−0.64 (−3.84 to 2.56)		−0.64 (−3.84 to 2.56)		−0.05 (−3.07 to 2.96)			−0.05 (−3.07 to 2.96)		−1.08 (−4.96 to 2.80)			−1.08 (−4.96 to 2.80)	
			*P* value	.69		.69		.97			.97		.58			.58	
	**SF-12 PCS^h^ change from baseline**																
		LS mean (SE)		−0.59 (0.89)		−0.75 (0.86)		−2.38 (0.87)			−1.59 (0.87)		−2.08 (1.17)			−2.82 (1.26)	
		**Difference (study-provided psychoeducation vs UC)**															
			LS mean (95% CI)	0.16 (−2.29 to 2.60)		0.16 (−2.29 to 2.60)		−0.79 (−3.23 to 1.64)			−0.79 (−3.23 to 1.64)		0.74 (−2.67 to 4.15)			0.74 (−2.67 to 4.15)	
			*P* value	.90		.90		.52			.52		.67			.67	
**Patient outcomes**																	
	**IMR^i,j^ change from baseline**																
		LS mean (SE)		1.12 (0.75)		1.04 (0.72)		4.34 (0.89)			3.40 (0.85)		4.47 (0.81)			3.61 (0.83)	
		**Difference (study-provided psychoeducation vs UC)**															
			LS mean (95% CI)	0.09 (−1.97 to 2.14)		0.09 (−1.97 to 2.14)		0.94 (−1.50 to 3.38)			0.94 (−1.50 to 3.38)		0.86 (−1.45 to 3.16)			0.86 (−1.45 to 3.16)	
			*P* value	.93		.93		.45			.45		.46			.46	
	**CGI-S^k,l^ change from baseline**																
		LS mean (SE)		−0.18 (0.10)		−0.12 (0.10)		−0.24 (0.11)			−0.30 (0.11)		−0.30 (0.13)			−0.44 (0.13)	
		**Difference (study-provided psychoeducation vs UC)**															
			LS mean (95% CI)	−0.06 (−0.34 to 0.22)		−0.06 (−0.34 to 0.22)		0.06 (−0.25 to 0.37)			0.06 (−0.25 to 0.37)		0.13 (−0.22 to 0.49)			0.13 (−0.22 to 0.49)	
			*P* value	.66		.66		.69			.69		.46			.46	

^a^UC: usual care.

^b^IEQ: Involvement Evaluation Questionnaire.

^c^Higher scores on the IEQ indicate a higher caregiver burden.

^d^LS: least squares.

^e^SF-12: 12-item Short Form Health Survey.

^f^MCS: mental component summary.

^g^Higher scores on the SF-12 indicate better health.

^h^PCS: physical component summary.

^i^IMR: Illness Management and Recovery.

^j^Higher scores on the IMR indicate better recovery status.

^k^CGI-S: Clinical Global Impression of Severity.

^l^Higher scores on the CGI-S indicate higher symptom severity.

**Table 7:** Summary of secondary end points: decreases in health resource utilization (N=148).

Health resource use	Baseline, n (%)	6 months, n (%)	12 months, n (%)
Hospitalizations	46 (31)	10 (7)	6 (4)
Emergency department visits	72 (49)	17 (12)	17 (12)
Intensive outpatient treatment	11 (7)	3 (2)	0 (0)

**Table 8:** Decreases in health resource utilization.

Health resource utilization decrease from baseline to month 12	Study-provided psychoeducation group	Usual care group
Reductions in hospitalizations	62% to 21%	61% to 18%
Emergency department visits	41% to 12%	56% to 11%
Intensive outpatient treatment	6 to 0 patients	5 to 0 patients

#### Safety

Of the 148 patients, 84 (56.8%) reported at least 1 TEAE during the study (Multimedia Appendix 4). No TEAEs were considered to be related to study-specific procedures. In total, 3 deaths were reported (n=1, 33% suicide; n=1, 33% drug overdose; and n=1, 33% cerebral hemorrhage), all in the UC group; none were considered related to trial-specific procedures. Safety in the paliperidone palmitate group was consistent with the known safety profile of paliperidone palmitate in adults, with no new events identified [31-33].

## Discussion

### Principal Findings and Key Learnings

No differences were observed over the 12-month study period between the study-provided psychoeducation and UC groups in either patient outcomes (TFs such as relapse, illness management, and change in clinical functioning) or caregiver outcomes (burden and physical and mental health functioning), with both groups showing significant improvement. This study aimed to fill the gap in the evidence base for FP by providing information on the effects of FP delivered specifically to caregivers using a telehealth-based platform. FP programs share several common characteristics but can vary considerably in length, setting, and content [34]. Although the results of this study did not show a benefit of the FP intervention at the level of exposure reached, consideration of the study limitations and additional key insights is important for the continued development of efficacious telehealth FP interventions.

Studies of caregiver-directed psychosocial interventions with positive outcomes have typically been longer (mean 57 weeks) and have provided more overall sessions (mean 28 sessions) than this study [24]. The duration of the study-provided psychoeducation program was also shorter than the minimum duration of 9 months recommended for FP by some experts [8,34]. However, other factors may have also played a role in the null results. Among the caregivers assigned to the study-provided psychoeducation group, there was a moderate amount of module completion, with 55% (40/73) of caregivers receiving >8 sessions. Although 16% (12/73) of caregivers received either 15 or 16 sessions of the intervention, 26% (19/73) of caregivers received either 0 or 1 session. The findings of exploratory analyses suggest that the wide range of participation in study-provided psychoeducation may have limited our ability to detect group differences. Furthermore, for caregivers who were engaged in study-provided psychoeducation, the psychoeducational modules that focused on relapse prevention, schizophrenia, and treatment adherence were received by <50% of caregivers despite the relevance of these topics to coping with a recent TF experienced by a family member. Therefore, limited participation in the study-provided psychoeducation and limited attention to psychoeducation about relapse prevention might have resulted in caregivers receiving insufficient information to avert events such as relapses and hospitalizations.

Most published studies on FP have evaluated models that include patients in the intervention. Since the inception of FP in the 1970s, several models have evolved to meet the needs of families, including FP and support [35,36], behavioral family therapy [37], and multi-family group therapy [38]. Studies of in-person family- and caregiver-focused psychoeducation programs have shown significant benefits over UC [6,7,24]. A meta-analysis of 18 randomized controlled trials of caregiver-directed psychosocial interventions for schizophrenia demonstrated significant improvements compared with UC in hospitalizations, relapse, and other patient outcomes, including visits to emergency departments, suicide attempts, and deaths [24]. A meta-analysis of 21 randomized controlled trials of interventions for informal caregivers found improved experiences of caring, increased quality of life, and reduced psychological distress among caregivers [7]. In the FIRST study, patients were not directly involved in the study-provided psychoeducation program, and caregivers were the primary focus. It is possible that the inclusion of both caregivers and patients in sessions has greater potential to improve outcomes over treatment with UC [19,20]. Furthermore, caregivers enrolled in the study-provided psychoeducation intervention were expected to identify their own educational needs and guide treatment by selecting most of the educational modules taught in the program. Research has shown that individuals often misjudge their knowledge or competence [39].

An unexpectedly large percentage of caregivers (54/148, 36.5%) discontinued participation in the study. The most common reasons for discontinuation were withdrawal of consent (17/148, 11.5%), others (17/148, 11.5%; which included administrative reasons [eg, lost to follow-up and nonadherence with study procedures] and personal reasons [eg, moved out of town and no longer serving as a caregiver]), lost to follow-up (13/148, 8.8%), and physician’s decision (5/148, 3.4%). Although caregiver demographic factors were similar between those who discontinued the study and those who completed the study, 80.6% (83/103) of caregivers who completed the study were parents of the patient compared with only 64% (29/45) of caregivers who dropped out. It is possible that the parents of patients may have been more committed and motivated to continue the study than caregivers who were not parents of the patient. In addition, per protocol, when a patient discontinued participation in the study, their caregivers were also discontinued. This may have also contributed to the high discontinuation rate among the caregivers.

The baseline characteristics of caregivers in the FIRST study may help to identify caregivers who are likely to sufficiently engage with a telehealth-based study-provided psychoeducation intervention and those who may need additional support to fully engage. In a post hoc analysis of the study-provided psychoeducation group comparing baseline characteristics of caregivers receiving ≤8 sessions with those receiving >8 sessions (Multimedia Appendix 3), caregivers who received >8 sessions were more likely to be older and parents of individuals with schizophrenia. Furthermore, except for the IEQ subscale score of *worrying*, the baseline IEQ total and subscale scores were lower among those who received >8 sessions, indicating lower caregiver burden. It is possible that caregivers with a higher burden may have been too distressed to engage in the program, regardless of the convenience of internet-based access to interventions, and dropped out early. As noted earlier, caregivers who discontinued participation within the first 12 months of the FIRST study were also more likely to be nonparent relatives with poorer health (Table 1). This finding may help future researchers develop strategies for adherence to treatment that may improve attendance, engagement, and continuous caregiver involvement.

Another limitation of this study was that the sample size was smaller than intended, which may have affected the ability to draw specific conclusions. In addition, patients were eligible for enrollment only if they had experienced at least one TF within 6 months of screening, indicating a high degree of clinical severity, and the observed TF rate in the FIRST study was higher than expected for comparable studies with similar sample sizes. The recovery period following a TF event (eg, psychiatric hospitalization) may be a particularly vulnerable period that requires an additional level of support not examined in this study to facilitate better outcomes. Furthermore, the median age of the patients in the FIRST study was 25.0 years, indicating that they were also early in the course of their illness. Typically, many patients have difficulty accepting their diagnosis [40] and experience high levels of stress, mood symptoms, and suicidal ideation during early illness [6]. The risk of relapse is very high during this period and can predict disease progression [6]. Implementing effective interventions early to prevent repeated relapses may reduce the associated decline in cognition and functioning [6].

The study-provided psychoeducation intervention was delivered across many study sites [31], which differed in the standard services provided for both the study-provided psychoeducation and UC groups. Another limitation of the implementation of the study-provided psychoeducation intervention is that the clinician provided by MyHealios was not a member of the treatment team; therefore, progress in the program was not integrated with patient care. This also precluded the ability of the clinician to relay potentially important clinical information learned from the caregiver to the treatment team about changes in the patient’s condition (eg, emergence of early signs of relapse and treatment nonadherence).

The results of this study coincide with a critical moment for telehealth interventions. Although telehealth interventions were only used by 8% of Americans in 2019, engagement with telehealth has grown dramatically in acceptance during the COVID-19 pandemic [41,42]. For example, in a community mental health authority in Michigan (Network180), the rates of telehealth services increased from 5% before the pandemic to 84% during the peak of the pandemic in 2020 [43]. In addition, many mental health professionals have recommended the ethical use of telehealth interventions to provide continued support and care to patients and caregivers throughout the pandemic rather than in-person interventions, noting that telehealth support can be just as effective and may result in fewer missed visits [44-46]. Insights on best practices for web-based delivery of mental health interventions are critically needed, and new models are under development [47]. Further research using FP methods, taking the lessons learned from the FIRST study into account, is warranted.

### Conclusions

The findings from this study provide valuable insights into a supplemental telehealth-based FP provided in the treatment of patients with early-phase schizophrenia spectrum disorders receiving paliperidone palmitate or oral antipsychotic medication. Key insights include the potential importance of supporting sufficient caregiver engagement; communication between clinicians, patients, and family members regarding treatment plans; and ensuring a link between clinicians providing psychoeducation to patients and the rest of their treatment team. Future studies in which telehealth interventions include caregiver–patient sessions and multicaregiver group sessions are warranted [19]. Meanwhile, traditional methods of delivering FP to caregivers and patients with schizophrenia spectrum disorders continue to have great potential for reducing caregiver burden and improving patient outcomes. As more telehealth psychoeducation platforms become available, we anticipate a continued exploration of how to adapt these important support programs to telehealth, with the goal of increasing benefits to patients and families.

## Appendix

Multimedia Appendix 1 CONSORT-EHEALTH (Consolidated Standards of Reporting Trials of Electronic and Mobile Health Applications and Online Telehealth) checklist (version 1.6.1).

Multimedia Appendix 2 Number of psychoeducation sessions provided to caregivers in the study-provided psychoeducation group (N=148).

Multimedia Appendix 3 Caregiver demographics and baseline characteristics by number of study-provided psychoeducation sessions (≤8 vs >8; safety analysis set).

Multimedia Appendix 4 Summary of safety data.

## Abbreviations

AE: adverse event
CGI-S: Clinical Global Impression of Severity
FIRST: Family Intervention in Recent-Onset Schizophrenia Treatment
FP: family psychoeducation
IEQ: Involvement Evaluation Questionnaire
IMR: Illness Management and Recovery
MCS: mental health component score
PCS: physical component score
SAE: serious adverse event
SF-12: 12-item Short Form Health Survey
TEAE: treatment-emergent adverse event
TF: treatment failure
UC: usual care
