# Supplementary figures and images for: Telehealth-Based Psychoeducation for Caregivers: The Family Intervention in Recent-Onset Schizophrenia Treatment Study
Source: JMIR Ment Health. 2022 Apr 15;9(4):e32492. doi: 10.2196/32492 (PMC9055490; doi:10.2196/32492)

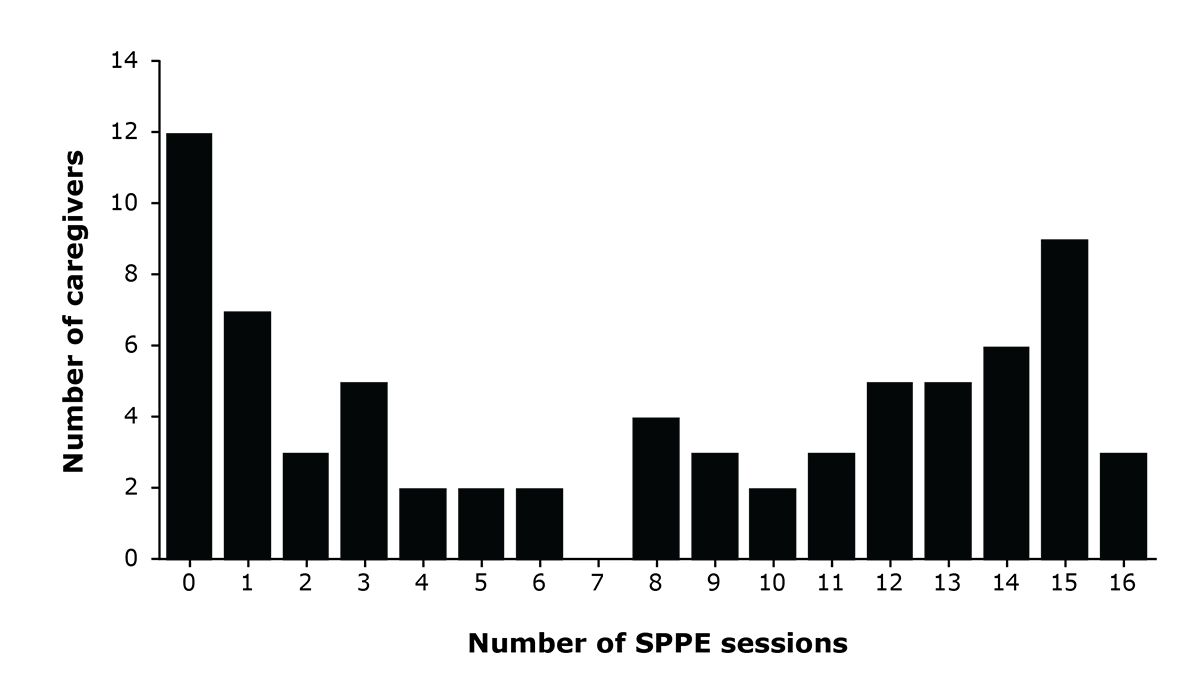

Supplement: Multimedia Appendix 2 [file mental_v9i4e32492_app2.png]
