# Supplementary material for: Telehealth-Based Psychoeducation for Caregivers: The Family Intervention in Recent-Onset Schizophrenia Treatment Study
Source: JMIR Ment Health. 2022 Apr 15;9(4):e32492. doi: 10.2196/32492 (PMC9055490; doi:10.2196/32492)
Supplement: Multimedia Appendix 3 [file mental_v9i4e32492_app3.docx]

**Multimedia Appendix 3**

Caregiver demographics and baseline characteristics by number of SPPE sessions (≤8 vs >8; safety analysis set).

| \|  \| Caregiver-focused SPPE and skills training group (n=73) \| \| \| \| \| --- \| --- \| --- \| --- \| --- \| \| n, (%) unless otherwise stated \| ≤8 sessions n=37 \| \| >8 sessions n=36 \| \| \|  \| n \| % \| n \| % \| \| Mean age, years (SD) \| 49.0 \| 11.2 \| 55.3 \| 10.3 \| \| Sex \|  \|  \|  \|  \| \| Male \| 9 \| 24 \| 5 \| 14 \| \| Female \| 28 \| 76 \| 31 \| 86 \| \| Race \|  \|  \|  \|  \| \| White \| 20 \| 54 \| 27 \| 75 \| \| Black or African American \| 13 \| 35 \| 5 \| 14 \| \| Multiple/other/not reported/unknown \| 4 \| 11 \| 4 \| 11 \| \| Ethnicity \|  \|  \|  \|  \| \| Hispanic or Latino \| 6 \| 16 \| 3 \| 8 \| \| Not Hispanic or Latino \| 31 \| 84 \| 33 \| 92 \| \|  \|  \|  \|  \|  \| \| Relationship with patient \|  \|  \|  \|  \| \| Parent \| 24 \| 65 \| 32 \| 89 \| \| Spouse/partner \| 6 \| 16 \| 0 \| 0 \| \| Other relative \| 4 \| 11 \| 1 \| 3 \| \| Friend \| 1 \| 3 \| 2 \| 6 \| \| Sibling \| 1 \| 3 \| 1 \| 3 \| \| Other \| 1 \| 3 \| 0 \| 0 \| \| IEQ, mean score (SD) \|  \|  \|  \|  \| \| Total \| 31.5 \| 19.2 \| 30.0 \| 13.1 \| \| Tension \| 8.2 \| 6.0 \| 8.1 \| 4.7 \| \| Supervision \| 3.5 \| 4.1 \| 2.7 \| 2.2 \| \| Worrying \| 10.8 \| 6.4 \| 11.9 \| 6.0 \| \| Urging \| 11.6 \| 6.5 \| 9.7 \| 5.1 \| \| SF-12 PCS, mean score (SD) \| 49.3 \| 11.3 \| 51.4 \| 9.1 \| \| SF-12 MCS, mean score (SD) \| 45.2 \| 10.2 \| 45.4 \| 10.7 \|   IEQ: Involvement Evaluation Questionnaire; MCS: mental component summary; PCS: physical component summary; SD: standard deviation; SF-12: 12-item Short-Form Health Survey; SPPE: study-provided psychoeducation. |  |  |  |  |
| --- | --- | --- | --- | --- | --- | --- | --- | --- | --- | --- | --- | --- | --- | --- | --- | --- | --- | --- | --- | --- | --- | --- | --- | --- | --- | --- | --- | --- | --- | --- | --- | --- | --- | --- | --- | --- | --- | --- | --- | --- | --- | --- | --- | --- | --- | --- | --- | --- | --- | --- | --- | --- | --- | --- | --- | --- | --- | --- | --- | --- | --- | --- | --- | --- | --- | --- | --- | --- | --- | --- | --- | --- | --- | --- | --- | --- | --- | --- | --- | --- | --- | --- | --- | --- | --- | --- | --- | --- | --- | --- | --- | --- | --- | --- | --- | --- | --- | --- | --- | --- | --- | --- | --- | --- | --- | --- | --- | --- | --- | --- | --- | --- | --- | --- | --- | --- | --- | --- | --- | --- | --- | --- | --- | --- | --- | --- | --- | --- | --- | --- | --- | --- | --- | --- | --- | --- | --- | --- | --- | --- | --- | --- | --- | --- | --- | --- | --- | --- | --- | --- | --- | --- | --- | --- |

|  |  |  |  |  |
| --- | --- | --- | --- | --- |
