# Supplementary material for: Telehealth-Based Psychoeducation for Caregivers: The Family Intervention in Recent-Onset Schizophrenia Treatment Study
Source: JMIR Ment Health. 2022 Apr 15;9(4):e32492. doi: 10.2196/32492 (PMC9055490; doi:10.2196/32492)
Supplement: Multimedia Appendix 4 [file mental_v9i4e32492_app4.docx]

**Multimedia Appendix 4**.

**Summary of safety data.**

|  | SPPE group  n = 73 | UC group  n = 75 |
| --- | --- | --- |
| ≥1 TEAE^a^, n (%) | 39 (53%) | 45 (60%) |
| Serious TEAEs, n (%) | 24 (33%) | 25 (33%) |
| Discontinuation due to TEAE | 1 (1%) | 0 |
| Death due to TEAE | 0 | 2 (3%)^b^ |

SPPE: study-provided psychoeducation; TEAE: treatment-emergent adverse event; UC, usual care.

^a^TEAEs that occurred in ≥5% of patients overall (n=148) were psychotic disorder and worsening schizophrenia (both 10%) and aggression, auditory hallucinations, and suicidal ideation (each 6%). TEAEs that were reported more frequently (≥5%) in patients in the UC group than those in the SPPE group were psychotic disorders (13% vs 12%), auditory hallucinations (7% vs 0%), aggression (5% vs 3%), and anxiety (5% vs 0%). TEAEs that were reported more frequently (≥5%) in patients in the SPPE group than the UC group were worsening schizophrenia (11% vs 4%) and suicidal ideation (6% vs 4%).

^b^One patient died due to suicide and one due to accidental overdose. Note: one patient in the UC group died from cerebral hemorrhage.
